# Anomalous dynamics of intruders in a crowded environment of mobile obstacles

**DOI:** 10.1038/ncomms11133

**Published:** 2016-04-04

**Authors:** Tatjana Sentjabrskaja, Emanuela Zaccarelli, Cristiano De Michele, Francesco Sciortino, Piero Tartaglia, Thomas Voigtmann, Stefan U. Egelhaaf, Marco Laurati

**Affiliations:** 1 Condensed Matter Physics Laboratory, Heinrich Heine University, 40225 Düsseldorf, Germany; 2 CNR-ISC, Università di Roma ‘La Sapienza', Piazzale A. Moro 2, Roma 00185, Italy; 3 Dipartimento di Fisica, Università di Roma ‘La Sapienza', Piazzale A. Moro 2, Roma 00185, Italy; 4 Institut für Materialphysik im Weltraum, Deutsches Zentrum für Luft- und Raumfahrt (DLR), 51170 Köln, Germany; 5 Heinrich Heine University, Universitätsstraße 1, 40225 Düsseldorf, Germany; 6 División de Ciencias e Ingeniería, Universidad de Guanajuato, Loma del Bosque 103, León 37150, Mexico

## Abstract

Many natural and industrial processes rely on constrained transport, such as proteins moving through cells, particles confined in nanocomposite materials or gels, individuals in highly dense collectives and vehicular traffic conditions. These are examples of motion through crowded environments, in which the host matrix may retain some glass-like dynamics. Here we investigate constrained transport in a colloidal model system, in which dilute small spheres move in a slowly rearranging, glassy matrix of large spheres. Using confocal differential dynamic microscopy and simulations, here we discover a critical size asymmetry, at which anomalous collective transport of the small particles appears, manifested as a logarithmic decay of the density autocorrelation functions. We demonstrate that the matrix mobility is central for the observed anomalous behaviour. These results, crucially depending on size-induced dynamic asymmetry, are of relevance for a wide range of phenomena ranging from glassy systems to cell biology.

## Abstract (web summary)

The classical Lorentz gas model is widely used to describe constrained transport, but its assumption of an immobile environment is not applicable to many biological and industrial processes. Here, the authors show that the mobility of the matrix induces anomalous, logarithmic dynamics of the confined particles.

## Graphical abstract

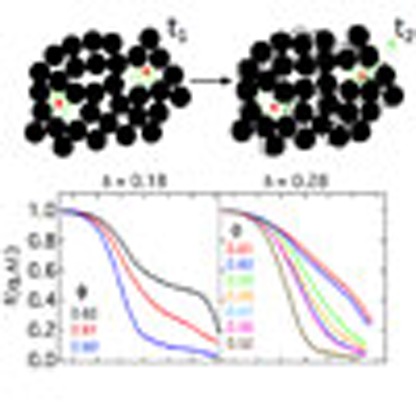

In the presence of a confining medium, the transport of objects deviates from normal diffusion. Anomalous behaviour, usually manifested by the presence of sub-diffusivity^1^,^2^, emerges as a common feature of the dynamics. In the Lorentz gas^3^,^4^, the prototype model for anomalous transport, point-like intruders move in voids between immobile, randomly-distributed particles. Their motion becomes sub-diffusive once the voids are barely interconnected. When a critical density of immobile particles is reached, they percolate and the intruder becomes localized^3^. Softness of the immobile particles or interactions among the intruders are known to modify this picture^5^,^6^,^7^,^8^,^9^,^10^.

So far the slow movement of the host matrix has been largely ignored, despite representing realistic situations of biological^11^,^12^,^13^,^14^,^15^,^16^ and industrial interest^17^,^18^,^19^,^20^,^21^,^22^,^23^. To address confined transport in slowly moving matrices, here we investigate a binary colloidal mixture of small and large hard spheres, of diameters *σ*_s_ and *σ*_l_, which represent intruders and host matrix, respectively. Changing the size ratio *δ*=*σ*_s_/*σ*_l_ we also modify the dynamic asymmetry of the system. We focus on volume fractions of large particles *ϕ*_*l*_>0.5 approaching the glass transition, occurring at 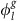
≈0.58. In contrast the volume fraction of the intruders *ϕ*_s_ is very small with *x*_s_≡*ϕ*_s_/*ϕ*=0.01. Such a system combines the confinement of a dilute fluid of mobile intruders with the slow dynamics of the matrix (Fig. 1a). It thus provides the simplest minimal model for the investigation of motion in crowded soft and biological matter.

Despite its conceptual simplicity, experimental investigations of the dynamics of small intruders in mixtures of Brownian particles with large size-asymmetry are scarce. This might be due to limitations in the spatial and temporal resolution of confocal microscopy which make it difficult to track particles that are significantly smaller than another species of Brownian, that is, at most micron-sized, particles. To overcome these limitations, we keep the selectivity of fluorescent labelling (Fig. 1b), which allows us to separately determine the small and large particles. However, instead of tracking we employ the recent Differential Dynamic Microscopy (DDM) technique^24^,^25^,^26^. This is based on the time correlation in Fourier space of the difference between images separated by a time delay Δ*t* (Fig. 1c) and provides a measure of the (isotropic) collective intermediate scattering function or density autocorrelation function *f*(*q*, Δ*t*), where *q* is the modulus of the wave vector **q** (Fig. 1d). The decay of *f*(*q*, Δ*t*) as a function of time delay Δ*t* corresponds to the loss of correlation of the particle density on a length scale determined by *q*^−1^ within the time delay Δ*t*. The decay time is therefore related to the characteristic time of the particle motions on the length scale *q*^−1^. Approaches similar to DDM, like fluorescence correlation spectroscopy, do not provide information on the probed length scale. This information is crucial to investigate the effect on the dynamics of the size of the voids in which the small particles move. The function *f*(*q*, Δ*t*) can also be obtained by dynamic light scattering, which, however, does not allow us to distinguish the two species by fluorescent labeling. We also study the same system by mode coupling theory (MCT) of the glass transition and, both in the case of mobile and immobile matrix particles, by numerical simulations, complementing the experimental results and providing insights on the underlying microscopic mechanisms. We observe anomalous dynamics of the small spheres at a critical size ratio *δ*_c_ and we show that this dynamical behavior is intimately connected to the slow dynamics of the matrix of large particles.

## Results

### Small particle dynamics

Figure 2a–d shows the measured collective intermediate scattering functions *f*(*q*, Δ*t*) of the small particles for size ratios *δ*=0.18 (Fig. 2a,c) and *δ*=0.28 (Fig. 2b,d) for different *ϕ* and *q*. For *δ*=0.18 and all *ϕ* and *q*, *f*(*q*, Δ*t*) versus Δ*t* shows an initial decay, followed by a *ϕ*-dependent intermediate plateau, and eventually a decay to zero at longer times (Fig. 2a). The initial decay can be associated with the Brownian motion of small particles within the voids of the large particles matrix. It becomes increasingly slower for increasing *ϕ* (Fig. 2a) and decreasing *q*, which means increasing length scale (Fig. 2c). The intermediate plateau indicates the dynamical arrest of the collective dynamics, that is, of density fluctuations, and hence the absence of diffusion on the length scale determined by *q*^−1^. The height of the plateau increases progressively with increasing *ϕ*, similarly to the scenario, in which a percolation-type transition is approached^5^,^27^, and indicates that voids become smaller and particles are increasingly localised^28^. The final decay to zero of *f*(*q*, Δ*t*) shows that particles are still able to diffuse at long times. For a larger size ratio, *δ*=0.28, and comparable *ϕ* values, a completely different scenario appears. Beyond *ϕ*≈0.60, *f*(*q*, Δ*t*) shows remarkable anomalous dynamics, manifested in an extended logarithmic decay over three decades in time. This intriguing behavior is mostly visible at *ϕ*⪞0.60 and *qσ*_l_≈3.5, that is, when probing a length scale of about 2*σ*_l_ (Fig. 2b), which is comparable with the size of the matrix particles.

The experimental findings are confirmed by simulations. For *δ*=0.20 no anomalous behavior of the small particles is detected in the collective *f*(*q*, Δ*t*) (Fig. 2e,g) and in the self *f*^self^(*q*, Δ*t*) correlation functions (Fig. 2i). Note that for *δ*=0.20, *f*(*q*, Δ*t*) displays a two step-relaxation and the presence of localisation (Fig. 2e,g), which is absent in *f*^self^(*q*, Δ*t*) (Fig. 2i). Also the mean squared displacements (MSD) 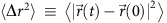, with 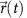 the position of a particle at time *t*, show almost no localisation at all *ϕ* (Fig. 2k). This decoupling between collective (*f*(*q*, Δ*t*)) and self dynamics (*f*^self^(*q*, Δ*t*), MSD) originates from the glassy environment in which the intruders move. Correlated motions of a group of intruders distributed within the matrix are more influenced by the slow dynamics of the matrix particles than uncorrelated single particle motions, which are mostly sensitive to the local structure of the voids^5^,^29^. For *δ*=0.35 we find the emergence of logarithmic anomalous relaxations of *f*(*q*, Δ*t*) (Fig. 2f,h) and *f*^self^(*q*, Δ*t*) (Fig. 2j), for comparable *q* as in the experiments. Additional simulations for *δ*=0.30 and *δ*=0.40 also show a logarithmic decay over a smaller time window. Furthermore, for *δ*=0.35 and *ϕ*⪞0.60 the MSD displays a clear sub-diffusive behavior, i.e., 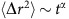 with *α*<1 (Fig. 2l). Finally, for *δ*=0.5, *f*(*q*, Δ*t*) and *f*^self^(*q*, Δ*t*) show a two-step decay and the MSD a localisation plateau at large *ϕ*, consistent with a standard glass transition of the small particles. At all investigated *δ* and for *ϕ*_*l*_>0.55, the dynamics of the large particles are very slow and at intermediate times are indicating localisation and motion within nearest neighbour cages of approximate size 0.1*σ*_l_ (Supplementary Fig. 1 and Supplementary Note 1).

These results suggest the existence of a critical size ratio *δ*_*c*_≃0.35 at which pronounced anomalous dynamics mark the transition from a diffusive to a glassy regime of the small particles moving in the large particles matrix. The *δ*_*c*_ and *ϕ* values where this transition is observed are slightly smaller in the experiments than in the simulations. This is attributed to the fact that in the experiments small particles are polydisperse, while in the simulations they are monodisperse. Polydispersity is expected to affect the transition since the average size particles might still be able to diffuse through the void spaces in the matrix, whereas the largest particles of the size distribution might no longer be able to diffuse through them. The crossover observed at *δ*_*c*_ is analogous to the transition from a diffusive to a localized state in models with fixed obstacles. However, the excluded volume of the intruder generates a coupling with the host matrix and, due to the mobility of the matrix, also between intruders in different voids, mutating localization into a glass transition due to the (slow) mobility of the matrix particles. Although this is apparently similar to intruders in a fixed matrix^5^,^6^,^7^, the logarithmic decay of *f*(*q*, Δ*t*) stands out as a novel feature.

On the basis of MCT, the appearance of logarithmic decays in *f*(*q*, Δ*t*)^30^,^31^,^32^ is usually attributed to competing collective arrest mechanisms, like caging and bonding, and to higher-order glass transition singularities^29^,^33^,^34^,^35^. We solved MCT equations for a binary mixture of hard spheres and *x*_s_=0.01. The resulting correlators *f*(*q*, Δ*t*) for a range of packing fractions around the MCT glass transition, *ϕ*_c_≈0.516 and *δ*=0.20 and 0.35, are shown in Fig. 3. No clear sign of logarithmic decay of *f*(*q*, Δ*t*) is found for these states in MCT: while an approximate logarithmic dependence of the decay is observed at *δ*=0.35, *ϕ*=0.51 and *qσ*_l_=3.4, this extends over an interval of times much shorter than in experiments and simulations. In addition, upon further increasing *ϕ* the logarithmic dependence does not take over, but instead a two-step decay is found, followed by the arrest of the dynamics. Indeed higher-order singularities are not present in this region of *ϕ* and *x*_s_ values^29^. On the other hand, the MSD obtained from MCT shows the qualitative signatures found in simulations: for *δ*=0.20<*δ*_c_, the long-time diffusion barely slows down with increasing *ϕ*, indicating a partially frozen glass in which the small particles are mobile. For *δ*=0.35≈*δ*_c_, anomalous sub-diffusion is observed, indicating that the glass-transition of the large particles and the localization transition of the small particles are close to each other. Thus, the appearance of approximately logarithmic decay in Fig. 3 could be a signal of the transition from coupled dynamics of the two species at large *δ* to decoupled dynamics at small *δ*.

### Void space explored by small particles

**Figure 1:**
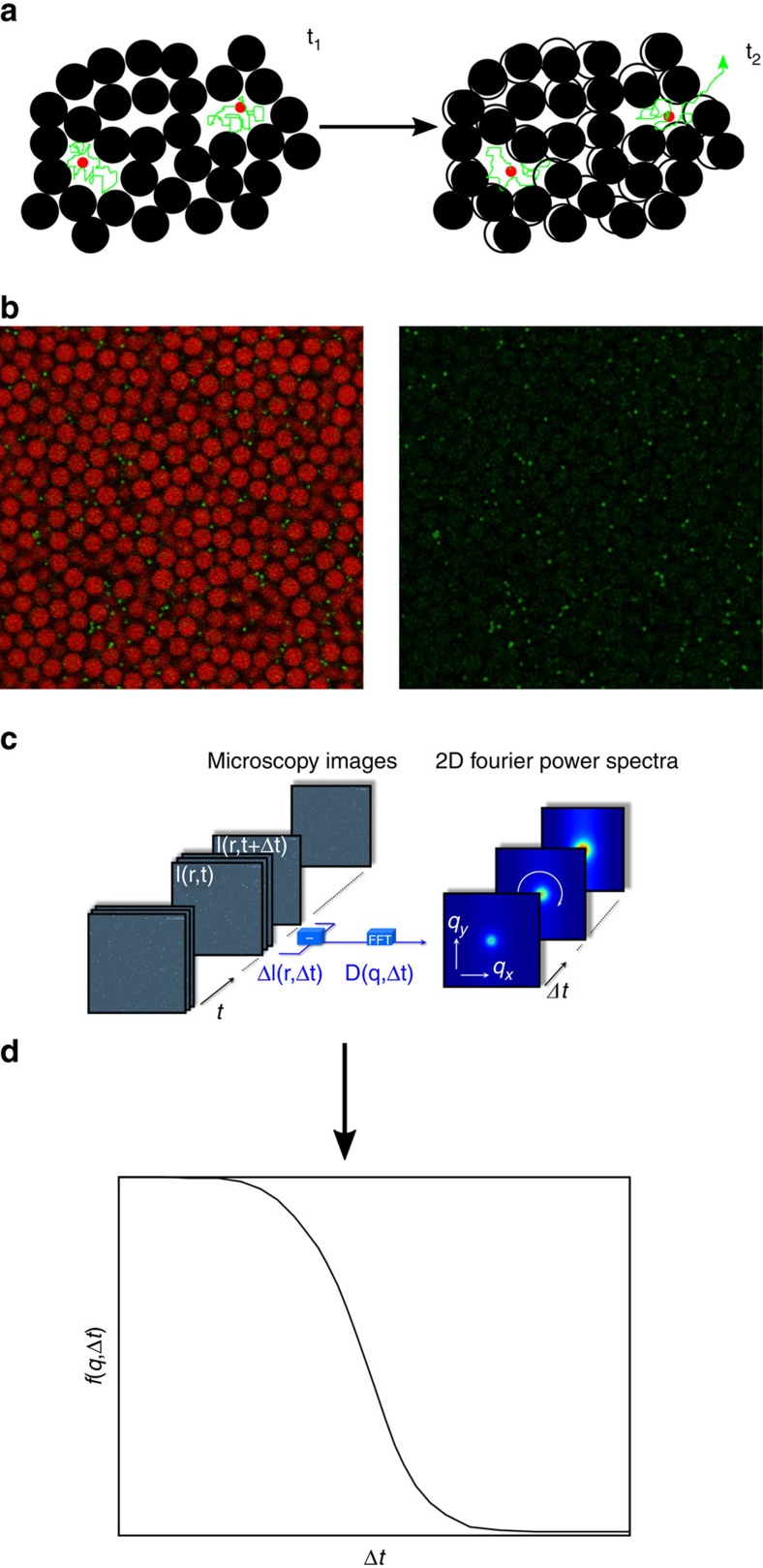
Illustration of the system and measurement method. (**a**) Schematic illustration of our system at two times *t*_1_ and *t*_2_>*t*_1_ highlighting the trajectories (green lines) of the intruders (red beads) in voids and between voids made possible due to the mobility of the matrix particles. (**b**) An exemplary confocal microscopy image of a mixture with *δ*=0.18 and *ϕ*=0.58 in which (left) both particles and (right) only the small particles are shown. (**c**) Image differences Δ*I*(*r*,Δ*t* ) at different delay times Δ*t* are Fourier transformed to give 2D Fourier power spectra for different Δ*t*. (**d**) After azimuthal averaging and additional treatment the intermediate scattering function *f*(*q*, Δ*t*) is obtained.

**Figure 2:**
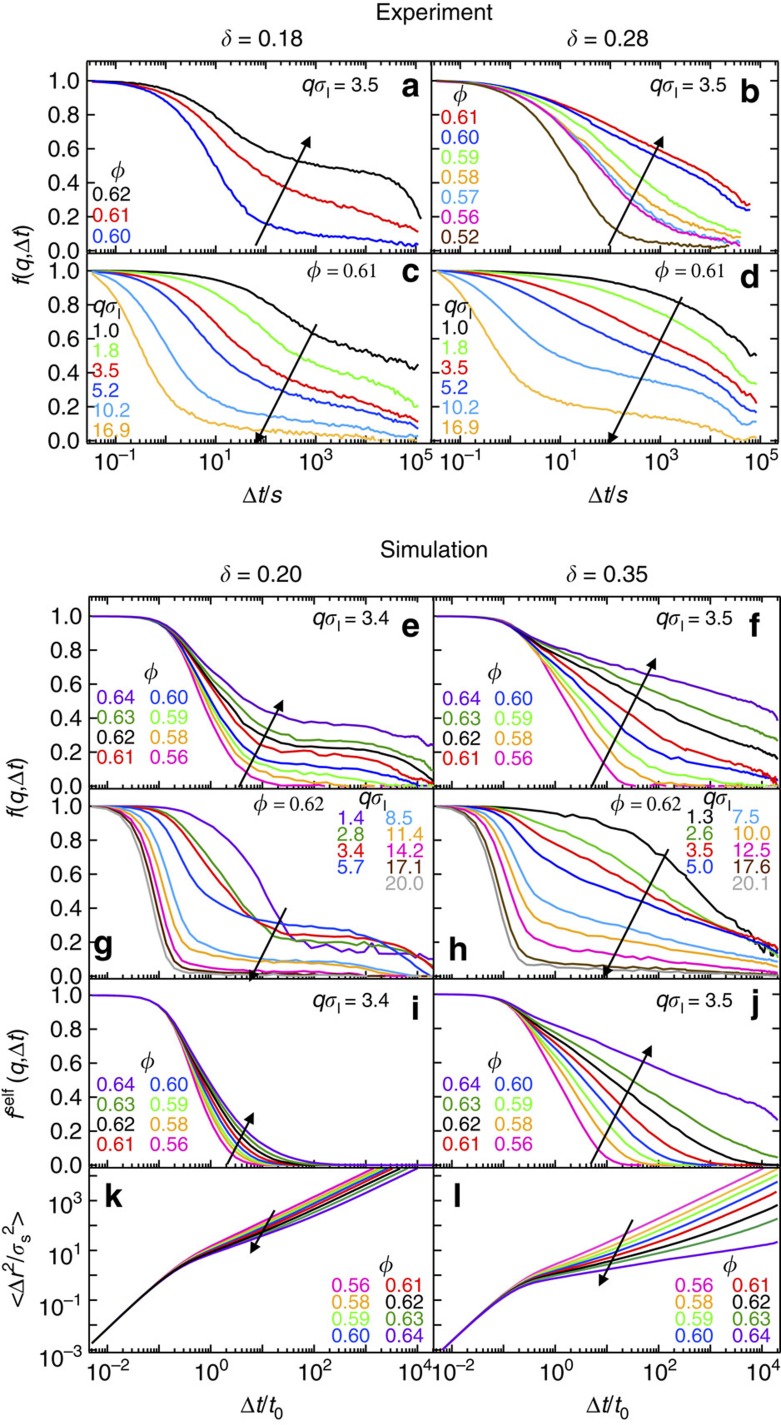
Dynamics of the intruders as observed in experiments and simulations. Collective *f*(*q*, Δ*t*) (**a**–**h**) and self *f*^*self*^(*q*, Δ*t*) (**i**–**j**) intermediate scattering functions and mean-squared displacements 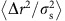 (**k**–**l**) as a function of delay time Δ*t*, describing the dynamics of small spheres in binary mixtures with size ratios *δ* below (left) and around (right) the onset of anomalous dynamics, for different magnitudes of the scattering vector *q* and total volume fraction *ϕ* (as indicated). Arrows indicate increasing *ϕ* and increasing *q* accordingly.

**Figure 3:**
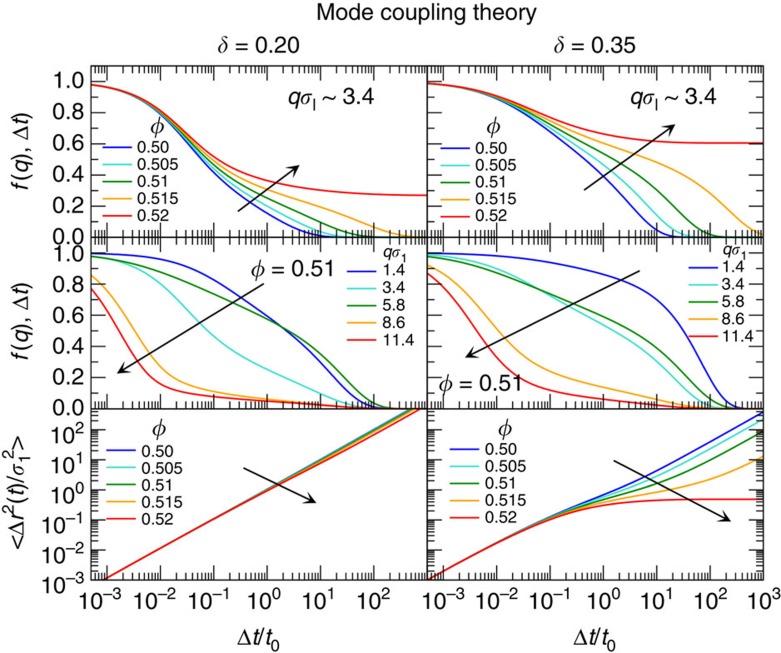
Dynamics of the intruders as predicted by MCT. Intermediate scattering functions *f*(*q*, Δ*t*) (top, middle) and mean-squared displacements 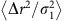 (bottom) describing the dynamics of small spheres in binary mixtures with size ratios delta below (left) and around (right) the onset of anomalous dynamics, for different magnitudes of the scattering vector *q* and total volume fraction *ϕ* (as indicated). Arrows indicate increasing *ϕ* or increasing *q* accordingly.

**Figure 4:**
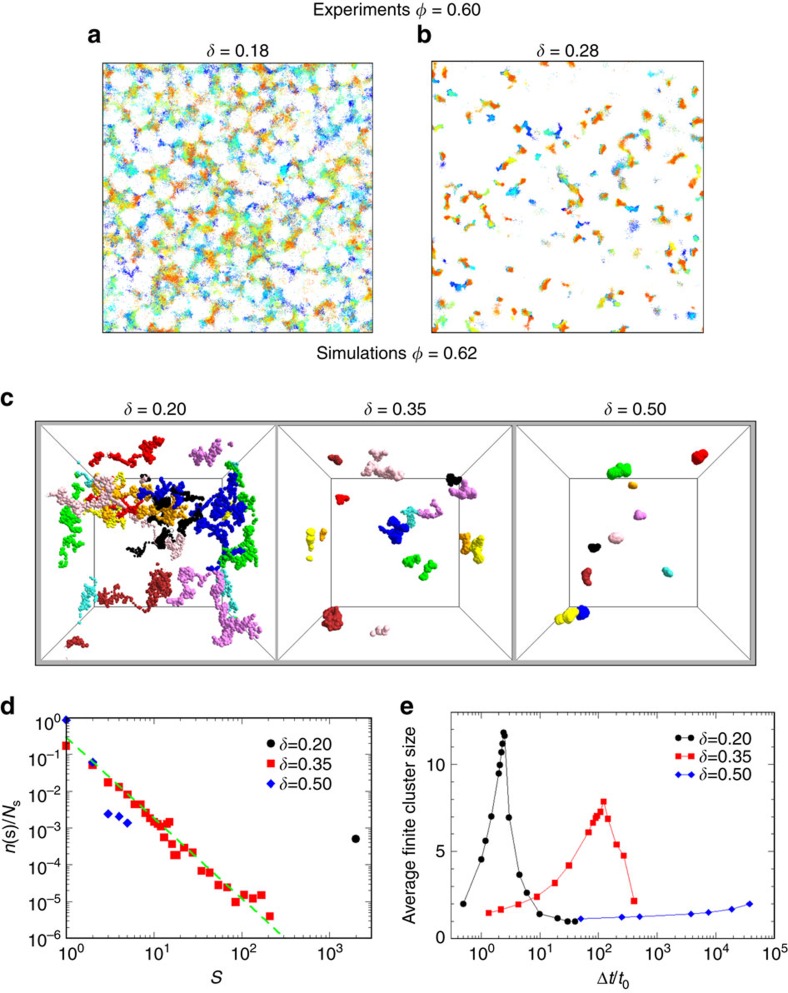
Illustration of the space explored by small particles during their motions. (**a**–**b**) Overlay of small particle positions at different times (colour coded from blue, corresponding to 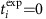 s to red, corresponding to 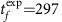 s with time steps of 33 s), obtained by particle tracking applied to 2D confocal microscopy images, for *ϕ*=0.60 and (**a**) *δ*=0.18, (**b**) *δ*=0.28. (**c**) Positions of 10 small particles (distinguished by different colours) for (left) *δ*=0.2, (middle) *δ*=0.35 and (right) *δ*=0.5, for a fixed total time of the trajectories 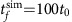, comparable with the experiments (**d**). Distribution *n*(*s*) (normalized by the number of small particles *N*_*s*_) of the size *s* of clusters as defined in Methods, providing a measure of the space explored by small particles, evaluated within a fixed time interval 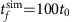. For *δ*=0.35 data follow a power-law dependence *n*(*s*)∼*s*^−2.19^, consistent with random percolation (dashed line), while for *δ*=0.20 all particles belong to the same cluster. (**e**) Average size *L*_c_ of finite clusters as a function of time, for different *δ*, as indicated. The maximum in each curve signals the onset of percolation.

A direct visualisation of small particle locations shows that the transition from diffusive dynamics at small *δ* to localised dynamics at large *δ* observed in experiments, simulations and theory is associated, similarly to models with immobile obstacles, with the transition from percolating to non-percolating voids within the matrix. However, a static picture of the void geometry cannot describe this transition, because the evolution of the void space involves a second timescale *t*_2_ (Fig. 1a, right) associated with the mobility of the matrix. To analyse the dynamic rearrangements of the void structure, we monitor the evolution of the position of the small particles which explore this evolving structure. Accordingly, in Fig. 4a,b we show superpositions of small particle locations in 2D time series of confocal images over a long total observation time 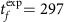 s, at which *f*(*q*, Δ*t*) for *δ*=0.18 shows a decay of correlations, while *f*(*q*, Δ*t*) for *δ*=0.28 is in the logarithmic regime. For *δ*=0.18 we find that, within the observation time, small particles easily explore the whole space of the accessible voids which form a percolating network. In contrast, for *δ*=0.28 particles mostly explore their local environment, since voids only barely connect even at long times, allowing only a slow, partial exploration of the available void space. Simulations provide not only particle locations but also single-particle trajectories in three dimensions allowing a more quantitative determination of the percolation of the explored space. Visualisations of typical small particle trajectories for a fixed observation time 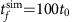 (comparable with the experiments) and three different values of *δ* confirm the experimental features (Fig. 4c): within the observation time small particles explore a percolated space for small *δ*, while for the critical size ratio the space is barely connected, indicating that particles can rarely escape the local environment which is only possible due to the stochastic opening and closing of channels between neighbouring void spaces, associated with the matrix motion on the long time scale t_2_. In addition, the simulations show that for even larger *δ* the explored space is disconnected. To quantify these observations we calculate the distribution *n*(*s*) of the size *s* of the clusters in which the space explored by small particles within a certain time interval is organized, as explained in Methods. The results are shown in Fig. 4d for different *δ* values for an observation time equal to 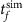. This time corresponds to the interval over which the cluster size distribution of the explored space for *δ*_*c*_ is close to percolation, as indicated by the power-law dependence *n*(*s*)∼*s*^−2.19^, consistent with random percolation predictions^36^. Percolation at 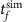 for *δ*_c_ is also indicated, in a finite-size system, by the maximum of the average size of finite-size clusters (excluding percolating clusters, calculated as explained in Methods) *L*_c_ as a function of time (Fig. 4e). For the other size ratios instead *L*_c_ is very small at 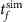. At small *δ* this is due to the fact that particles can easily move through channels connecting voids, and thus the explored space quickly associates into a percolating cluster. On the other hand, for large *δ* the creation of channels that allow the small particles to move between neighbouring void spaces is rare, and thus percolation of the explored space does not occur at 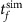 and only voids corresponding to the size of monomers, dimers and few-mers are observed. This analysis reveals very different timescales at which the explored space percolates at different *δ*. These timescales depend, besides *δ*, on the timescale *t*_2_ of the evolution of the void space, associated with the thermal motion of the matrix particles: yet this analysis is not offering substantial evidence that this mobility of the matrix is causing the logarithmic decays of the correlators observed at *δ*_c_.

### Comparison between mobile and immobile matrix

**Figure 5:**
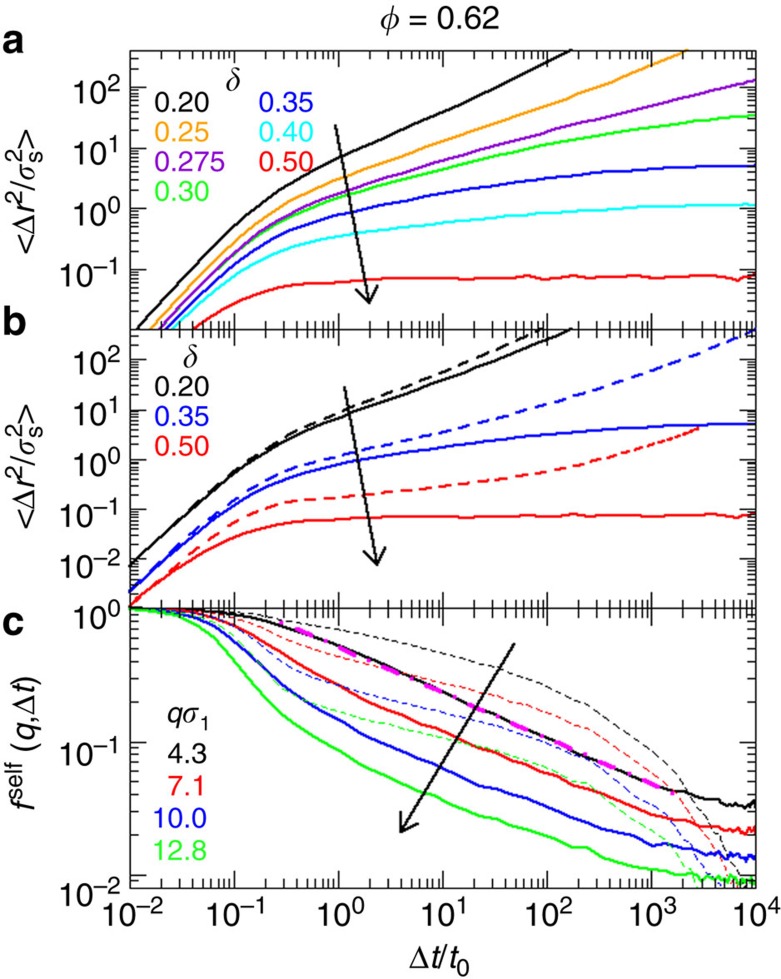
Small particle dynamics in a mobile or immobile large particles matrix. (**a**) MSDs of the small particles for immobile large particles at *ϕ*=0.62 and various values of *δ*, as indicated. For 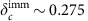 a clear subdiffusive behavior is observed at all times. (**b**) Comparison of the MSDs of the small particles at *ϕ*=0.62 for mobile (dashed lines) and immobile (full lines) large particles, for increasing *δ*, as indicated. (**c**) Self intermediate scattering functions *f*^self^(*q*, Δ*t*) at *ϕ*=0.62 and different wavevectors *qσ*_l_, as indicated, for *δ*=0.25 (immobile, full lines) and *δ*=0.35 (mobile, dashed lines) highlighting the power-law dependence (dot-dashed line) in the immobile case. Arrows indicate increasing Δ or increasing *q* accordingly.

To go one step further and link the residual mobility of the matrix particles with the anomalous logarithmic decays, we perform additional simulations (for *ϕ*=0.62) for immobile matrix particles and compare the dynamics of the intruders with the case of a mobile matrix. When the large particles are immobile (Fig. 5a), the MSD shows a sub-diffusive regime (MSD∼*t*^α^) followed by diffusion at long times (upward curvature) or localization (downward curvature), depending on *δ*. The crossover between these two long time behaviors takes place at a critical size ratio 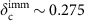 where the MSD remains subdiffusive also at long times^2^. The value of *δ*_*c*_ is smaller for the simulation with immobile large particles. This finding is consistent with the opening of channels as a consequence of the thermal motion of the matrix particles. In the case of mobile matrix particles localisation is *never* observed (Fig. 5b): even for large *δ*, the residual motion of the matrix allows the small particles to move and hence their MSD increases at long times. Furthermore, the subdiffusive regime observed in the case of an immobile matrix is only observed for 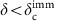 and thus in a smaller range than for mobile particles. This is consistent with the opening of channels as a consequence of the thermal motion of the matrix particles, which allows larger particles to move between voids. We also find that *f*^self^(*q*, Δ*t*) calculated for the case of an immobile matrix displays a power-law dependence on time extending for several decades (Fig. 5c), as also observed in the Lorentz gas model^37^, while the collective *f*(*q*, Δ*t*) displays neither a power-law nor a logarithmic dependence (Supplementary Fig. 2 and Supplementary Note 2). In the case of a mobile matrix, however, power law behaviour is not observed but, close to *δ*_c_, a logarithmic dependence is found. Thus, thermal motion of the matrix particles gives rise to the logarithmic decay, a novel type of dynamics which does not occur in models with immobile obstacles.

## Discussion

Our combined experimental, simulation and theoretical study shows that dynamics of intruders in a mobile crowded environment requires a description beyond that provided by models with a matrix of fixed obstacles. The novel application of the confocal DDM technique to concentrated binary colloidal mixtures allows us to investigate the collective dynamics of intruders in a mobile matrix, revealing extended anomalous dynamics for specific values of the size asymmetry and of the probed length scale. While the Lorentz model predicts a power-law behavior, which is typical for systems close to a percolation transition, in the case of a mobile matrix we observe a logarithmic decay of the collective and self density fluctuations over at least three decades in time, at length scales comparable to the size of the matrix particles. This logarithmic decay marks the transition between a diffusive behaviour of intruders in a glassy medium for small size ratios *δ*<*δ*_c_, where transient localization is due to the excluded volume of the mobile matrix, and glassy dynamics of the intruders at large size ratios *δ*>*δ*_c_, due to crowding. Our results thus show that both percolation and glassy dynamics have to be considered. By comparing mobile and immobile matrix environments, we demonstrate that the dynamics of the small particles is profoundly altered, in a qualitative way, by the continuous evolution of channels in the mobile matrix, due to the thermal motion of large particles. A mobile matrix corresponds to an environment in which small intruders move in many real systems and applications, like in glasses, nanocomposite materials, chromatography, catalysis, oil recovery, drug delivery or cell signaling, cell interiors, human and animal crowds and vehicular traffic. We thus expect that our findings will inspire the development of a more realistic description of these situations and will stimulate theoretical studies to refine the MCT predictions.

## Methods

### Materials

We investigated dispersions of sterically stabilized polymethyl methacrylate (PMMA) spheres of diameters 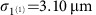 (polydispersity 0.07) or 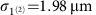 (polydispersity 0.07) mixed with spheres of diameter *σ*_s_=0.56 μm (polydispersity 0.13) (fluorescently labeled with nitrobenzoxadiazole), in a cis-decalin/cycloheptyl-bromide mixture which closely matches their density and refractive index. The size ratio of the mixtures is 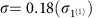 and 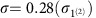, respectively. After adding salt (tetrabutylammoniumchloride), this system presents hard-sphere like interactions^38^,^39^. A sediment of the large spheres with *ϕ*=0.65 or of the small spheres with *ϕ*=0.67, as estimated from comparison with numerical simulations and experiments^40^,^41^, is diluted to obtain one-component dispersions with desired volume fraction *ϕ*. Following a recent study^42^, the uncertainty Δ*ϕ* can be as large or above 3%. Using the nominal volume fraction *ϕ* of the large spheres as a reference, the volume fraction of the samples containing the small particles are adjusted in order to obtain comparable linear viscoelastic moduli in units of the energy density 3*k*_B_*T*/4*πR*^3^, where *k*_B_ is the Boltzmann constant, *T* the temperature and *R* the particles' radius, while multiplying the frequency by the free-diffusion Brownian time 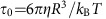, where *η*=2.2 mPa s is the solvent viscosity. In this way we obtain samples with comparable rheological properties and, according to the generalised Stokes–Einstein relation^43^, also dynamics and hence a similar location with respect to the glass transition. The comparable dynamics but different polydispersities of the one-component samples imply slightly different *ϕ*. Samples with different total volume fractions and a fixed composition, namely a fraction of small particles *x*_s_=*ϕ*_s_/*ϕ*=0.01, where *ϕ*_s_ is the volume fraction of small particles, are prepared by mixing the one-component samples.

### DDM measurements

Confocal microscopy images were acquired in a plane at a depth of approximately 30 μm from the coverslip. Images with 512 × 512 pixels, corresponding to 107 × 107 μm^2^, were acquired at a fast rate of 30 frames per second to follow the short-time dynamics and at a slow rate, between 0.07 and 0.33 frames per second, depending on sample, to follow the long-time dynamics. Image series were acquired using a Nikon A1R-MP confocal scanning unit mounted on a Nikon Ti-U inverted microscope, with a 60 × Nikon Plan Apo oil immersion objective (NA=1.40). The pixel size at this magnification is 0.21 × 0.21 μm. The confocal images were acquired with the maximum pinhole size allowed by the microscope, corresponding to a pinhole diameter of 255 μm. Time series of 10^4^ images were acquired for 2–5 different volumes, depending on sample.

### DDM analysis

Particle movements induce fluctuations of the fluorescence intensity in the images, *i*(*x*, *y*, *t*), with *x*, *y* the coordinates of a pixel in the image and *t* the time at which the image was recorded. To obtain additional information on the characteristic length scales of particle motions, *i*(*x*, *y*, *t*) can be Fourier transformed, yielding 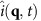, with **q** the wave vector in Fourier space, and then differences of the Fourier transformed image intensities can be correlated (Fig. 1c) to obtain the image structure function *D*(**q**, Δ*t*):


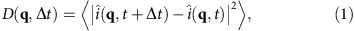


where 〈〉 represents an ensemble average. This analysis technique is named DDM^24^. The intermediate scattering function *f*(**q**, Δ*t*) (Fig. 1d) can be extracted from the image structure function:


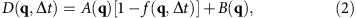


with 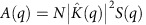, where *N* is the number of particles in the observed volume, 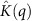 is the Fourier transform of the Point-Spread Function of the microscope, *S*(*q*) is the static structure factor of the system, and *B*(*q*) accounts for the camera noise. The inverse of the wave vector *q* determines the length scale over which the particle dynamics are probed. Thus *f*(**q**, Δ*t*) is obtained, similarly to dynamic light scattering (DLS)^44^, but for the present system the advantage of DDM over DLS is that fluctuations of the incoherent fluorescence signal can be correlated, a possibility which is excluded by the requirement of coherence of light in DLS. Furthermore, use of a confocal microscope drastically reduces the amount of background fluorescence of the measurements, significantly improving the determination of *f*(**q**, Δ*t*). The effect of particles moving in and out of the observation plane on *f*(**q**, Δ*t*) was found to be negligible for all samples, as determined by the q-dependence of the relaxation times of the initial decay of *f*(**q**, Δ*t*), where no plateau at small *q* values was observed^25^,^45^.

### Particle localization

Coordinates of the small particles were extracted from time series of 2-dimensional images using standard particle localization routines based on the centroiding technique^46^. Only the particle positions at each time could be determined, not the full trajectories. Indeed the displacement of small particles during the time delay Δ*t* between two successive frames is comparable or larger than their diameter, which implies that identifying particles after a Δ*t* becomes too uncertain.

### Simulations

We perform event-driven molecular dynamics simulations^47^ in the *NVT* ensemble in a cubic box with periodic boundary conditions for binary mixtures of hard spheres, of which the large components are 7% polydisperse by a discrete Gaussian distribution^48^ and the small ones are monodisperse. For each studied *δ* we vary the total number of particles in the range of a few thousands. The number of small particles thus varies from 1980 for *δ*=0.2 to 292 for *δ*=0.5. Mass and length are measured in units of particle mass *m*, average large particle diameter *σ*_l_, whereas time is in units of 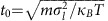, where *k*_*B*_ is the Boltzmann constant and *T* the temperature. For the simulations with immobile hard spheres, after equilibration of the mixture, we freeze the large particles only. To roughly estimate the critical size ratio which demarcates the transition between diffusive and localized states, we averaged results over ten different matrix realizations.

### Mode coupling theory

The equations determining *f*(*q*, *t*) and 〈Δ*r*^2^(*t*)〉 within MCT were solved for a binary mixture of hard spheres within the Percus–Yevick approximation for the static structure; for details on the theory and the numerical procedure, see ref. 29. The *f*(*q*, Δ*t*) were obtained using a wave-number grid of equidistant steps Δ*q*=0.4/*σ*_l_, with large-*q* cutoff *qσ*_l_=400. Brownian dynamics is assumed with the short-time diffusion coefficients following the Stokes–Einstein relation; the diffusion coefficient of the large particles sets the unit of time 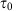. In the calculations, the total packing fraction *ϕ* is varied, keeping *x*_s_=*ϕ*_*s*_/*ϕ*=0.01 fixed.

### Calculation of the size distribution of the explored space

To evaluate the distribution of space sampled by the small particles during time we employ the following procedure: First we generate a sequence of *N*_c_ configurations saved at equally spaced times *t*_i_ (with i=1… *N*_c_) within a given time window 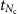. The time interval Δ*t*_c_ between two successive configurations, i.e., Δ*t*_c_=*t*_i+1_−*t*_i_ is chosen in such a way that 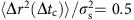. Second, we overlap all *N*_*c*_ configurations and perform a cluster size analysis according to the following criteria: (i) the same particle at different times *t*_*i*_ belong to the same cluster; (ii) if two particles overlap, they belong to the same cluster; (iii) the size *s* of a cluster is defined as the number of distinct particles belonging to the same cluster (running from one to the total number of small particles). To improve statistics we average the cluster size distribution *n*(*s*) over a set of at least 10 independent groups of *N*_*c*_ configurations. The average size of finite clusters is calculated as *L*_c_=∑ *s*^2^*n*(*s*)/∑ *sn*(*s*), excluding percolating clusters.

## Additional information

**How to cite this article:** Sentjabrskaja, T. *et al*. Anomalous dynamics of intruders in a crowded environment of mobile obstacles. *Nat. Commun.* 7:11133 doi: 10.1038/ncomms11133 (2016).

## Supplementary Material

Supplementary Information Supplementary Figures 1-2, Supplementary Notes 1-2 and Supplementary References
