# Supplementary material for: Anomalous dynamics of intruders in a crowded environment of mobile obstacles
Source: Nat Commun. 2016 Apr 4;7:11133. doi: 10.1038/ncomms11133 (PMC4822008; doi:10.1038/ncomms11133)
Supplement: Supplementary Information — Supplementary Figures 1-2, Supplementary Notes 1-2 and Supplementary References [file ncomms11133-s1.pdf]

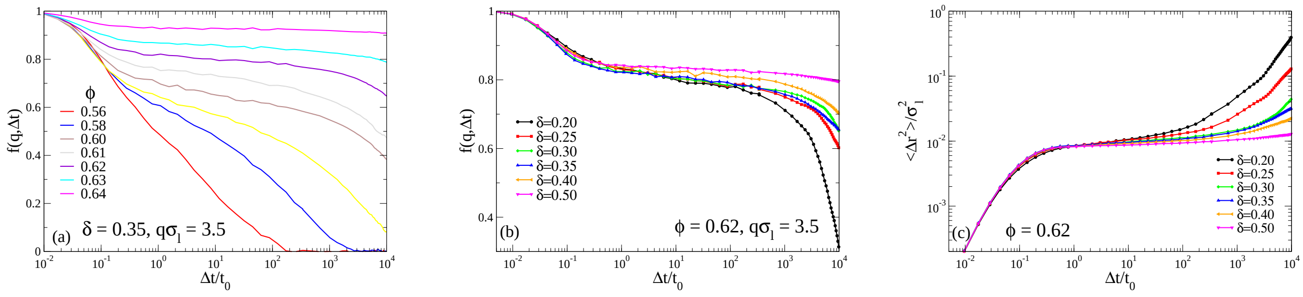

Supplementary Figure 1. Large particle dynamics as obtained in simulations Collective intermediate scattering functions $f(q,\Delta t)$ at $q\sigma_l \sim 3.5$ for (a) $\delta = 0.35$, and different ϕ (as indicated) and (b) for $\phi = 0.62$ and different δ (as indicated) (c) MSD for $\phi = 0.62$ and different δ (as indicated).

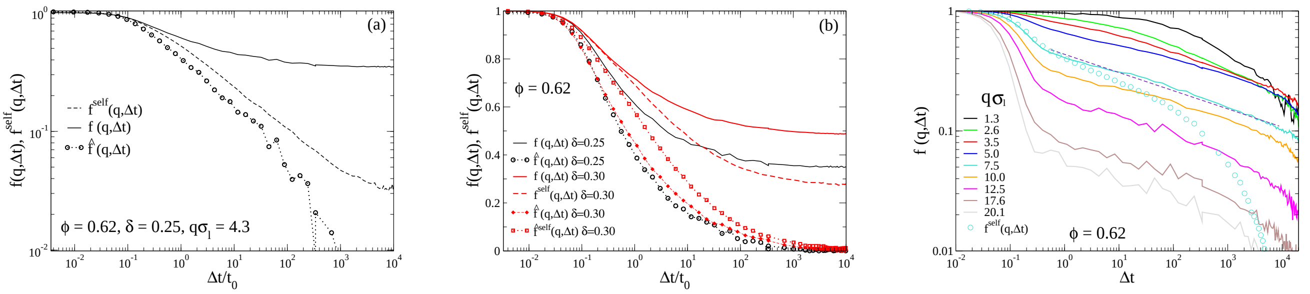

Supplementary Figure 2. Small particle dynamics in an immobile matrix (a) Collective (full curve), self (dashed curve) and scaled $\hat{f}(q,\Delta t)$ (see text, circles) intermediate scattering functions for $\phi = 0.62$, $\delta = 0.25$ and $q\sigma_l \simeq 4.3$ in log-log plot; (b) collective (full curve), self (dashed curve) and scaled $\hat{f}(q,\Delta t)$ (symbols) for $\phi = 0.62$ and $\delta = 0.25, q\sigma_l \simeq 4.3$ (black), $\delta = 0.30, q\sigma_l \simeq 3.7$ (red) in semi-log plot; (c) Same data as in Fig.2h of the manuscript, but in log-log scale instead of semi-log scale: collective correlators for $\phi = 0.62$ for various wavevectors.

Supplementary Notes

Supplementary Note 1: Large particle dynamics

The small particle dynamics display a dramatic change of behaviour at the critical size ratio, which can be associated with changes in the mechanism of arrest and the transition from caging at large δ to localisation at small δ, related to the decoupling of the dynamics of the two species. On the other hand the arrest mechanism of the large particles, caging by other large particles, is not significantly affected by the presence of the small fraction, $x_s = 0.01$, of small particles, irrespective of size ratio. As an example, Supplementary Fig.1(a) shows that large particles at $\delta_c = 0.35$, where the small particles show anomalous dynamics, approach a standard glass transition upon increasing ϕ, characterised by a typical two-step decay. Furthermore, Supplementary Fig.1(b) and (c) show that, upon changing δ, the localisation length, i.e. the cage size, does not change significantly, as evident from the plateau height of both the MSDs ($\sim 0.1\sigma_l^2$) and the density correlators. The cage though becomes more mobile with decreasing δ, as shown by the faster dynamics at long times, indicating an interesting coupling between the increased mobility of the small and large particles with decreasing δ. This coupling might be related to the fact that at small δ the small particles do not hinder the large particle movements due to their small size and large mobility.

Supplementary Note 2: Frozen vs. mobile matrix of large particles

Here we want to compare simulations of a fully mobile binary mixture of hard spheres and one where the large particles are immobile. For the latter situation, *quantitatively* accurate results can only be obtained when one considers a large system size and also performs an average over several matrix realizations, as done in previous works [2, 3, 4]. However, our aim is only to provide a *qualitative* comparison with the mobile case, for which our approach, based on a single realization for a system size of $O(10^3)$ particles, is sufficient, as indicated by the fact that the MSD for the immobile matrix case reported in Fig.5(a) displays a qualitative behavior which is compatible with that of the Lorentz gas[4].

Fig. 5(c) of the main article shows that the small particle self correlators for the immobile matrix case display (at intermediate time) a power-law behavior. It is to be noted that these correlators, even below the critical size ratio ~ 0.3, display a long-time finite value, i.e. a residual non-ergodicity. Indeed, different from studies on the Lorentz gas [5], we include among the intruders small particles trapped in finite size voids, i.e. not pertaining to the percolating cluster of voids, to make the analogy with the fully mobile case. The collective correlators, for the situation where the self ones show a power-law dependence on time, do not present the same behavior (Supplementary Fig.2(a)). Nevertheless, defining a scaled correlator $\hat{f}(q,\Delta t) = (f(q,\Delta t) - f(q,\infty))/(1 - f(q,\infty))$, which allows us to remove the contribution of the frozen-in component to the correlation function [6], we see that a power-law behavior seems to emerge also for the collective correlators, even though our current numerical resolution is not good enough to determine this clearly. However, the important point is that in semi-log plot (Supplementary Fig.2(b)) all correlators (self, collective and scaled) for frozen matrix conditions do not show a logarithmic decay in any time window or wavevector. Finally, in Supplementary Fig.2(c) the correlators for the mobile matrix at the critical size ratio are reported in log-log plot showing that at $q\sigma_l = 3.5$, where the anomalous logarithmic behavior is observed, a power-law decay cannot describe the data. It is interesting to note that at a larger value of $q\sigma_l = 7.5$ the data might approach this behavior at long times, even though within a two-step decay. The power-law exponent of about 0.5 is also close to the Lorentz gas (0.527) and to MCT predictions. This suggests that at the smaller length scales probed at larger q values, the particles mainly see the local environment and localisation, while only at smaller q values the network structure of voids is explored and leads to anomalous behavior. Note though that the self correlators significantly deviate from power-law behavior. In summary, these results complement those provided in the manuscript and show that small particles moving in a frozen matrix behave very differently from those moving in a glassy but mobile matrix of large particles.

Supplementary References

[1] Bosse, J. & Kaneko, Y. Self-diffusion in supercooled binary liquids. *Phys. Rev. Lett.* **74**, 4023–4026 (1995).

[2] Kurzidim, J., Coslovich, D. & Kahl, G. Single-particle and collective slow dynamics of colloids in porous confinement. *Phys. Rev. Lett.* **103**, 138303 (2009).

[3] Kim, K., Miyazaki, K. & Saito, S. Slow dynamics in random media: Crossover from glass to localization transition. *Europhys. Lett.* **88**, 36002 (2009).

[4] Höfling, F., Franosch, T. & Frey, E. Localization transition of the three-dimensional lorentz model and continuum percolation. *Phys. Rev. Lett.* **96**, 165901 (2006).

[5] Spanner, M., Schnyder, S. K., Hofling, F., Voigtmann, T. & Franosch, T. Dynamic arrest in model porous media-intermediate scattering functions. *Soft Matter* **9**, 1604–1611 (2013).

[6] Kertesz, J. & Metzger, J. Properties of the density relaxation function in classical diffusion models with percolation transition. *J. Phys. A: Math. Gen.* **16**, L735 (1983).
